# Bayesian Physics-Based Modeling of Tau Propagation in Alzheimer's Disease

**DOI:** 10.3389/fphys.2021.702975

**Published:** 2021-07-16

**Authors:** Amelie Schäfer, Mathias Peirlinck, Kevin Linka, Ellen Kuhl, The Alzheimer's Disease Neuroimaging Initiative (ADNI)

**Affiliations:** ^1^Department of Mechanical Engineering, Stanford University, Stanford, CA, United States; ^2^Institute of Continuum and Materials Mechanics, Hamburg University of Technology, Hamburg, Germany

**Keywords:** Alzheimer's disease, network diffusion model, tau PET, Bayesian inference, hierarchical modeling, uncertainty quantification

## Abstract

Amyloid-β and hyperphosphorylated tau protein are known drivers of neuropathology in Alzheimer's disease. Tau in particular spreads in the brains of patients following a spatiotemporal pattern that is highly sterotypical and correlated with subsequent neurodegeneration. Novel medical imaging techniques can now visualize the distribution of tau in the brain *in vivo*, allowing for new insights to the dynamics of this biomarker. Here we personalize a network diffusion model with global spreading and local production terms to longitudinal tau positron emission tomography data of 76 subjects from the Alzheimer's Disease Neuroimaging Initiative. We use Bayesian inference with a hierarchical prior structure to infer means and credible intervals for our model parameters on group and subject levels. Our results show that the group average protein production rate for amyloid positive subjects is significantly higher with 0.019±0.27/yr, than that for amyloid negative subjects with −0.143±0.21/yr (*p* = 0.0075). These results support the hypothesis that amyloid pathology drives tau pathology. The calibrated model could serve as a valuable clinical tool to identify optimal time points for follow-up scans and predict the timeline of disease progression.

## 1. Introduction

Alzheimer's disease currently affects one out of 10 adults over the age of 65 in the United States (Association, 2019). Due to demographic changes worldwide, the prevalence and public health impact of this neurodegenerative disease is projected to more than double in the next 30 years. Effective therapeutic interventions require early diagnosis and a detailed understanding of the early mechanisms driving pathology. For Alzheimer's disease, this poses a particular challenge since clinical diagnosis is currently possible only with the appearance of cognitive impairment at late disease stages. We now know that the first pathological changes which initiate the disease may happen up to decades before the presence of cognitive symptoms (Bateman et al., 2012; Jack et al., 2013). Investigating these early disease mechanisms is crucial, if we want to understand the timeline of disease progression and identify early access points for intervention.

It is well accepted that two proteins, amyloid-β and tau, play a major role in disease initiation and represent important biomarkers for disease progress (Duyckaerts et al., 2009). Amyloid and tau are both present in the healthy brain, but have been found to accumulate and aggregate in abnormal amounts and pathological forms in the brains of Alzheimer's patients. The amyloid hypothesis states that at the early stages of disease, amyloid-β starts to accumulate widely across the neocortex. Subsequently, hyperphosphorylated tau starts to accumulate and aggregate in neurofibrillary tangles in more and more areas of the brain, ultimately causing neurodegeneration and cognitive impairment (Jack and Holtzman, 2013). The sequence of when and where neurofibrillary tangles of tau emerge has been shown to follow a highly reproducible pattern. Cross-sectional autopsy studies have confirmed that tangles first appear in the transentorhinal and entorhinal cortex in early disease stages, then emerge in the neighboring hippocampus and regions of the temporal lobe, before ultimately spreading into more distantly connected areas of the neocortex (Braak and Braak, 1991; Braak et al., 2006). There is strong evidence from animal and imaging studies that hyperphosphorylated tau spreads intracellularly along axons in the brain (De Calignon et al., 2012; Liu et al., 2012; Jones et al., 2017; Pereira et al., 2019), explaining how the pathology propagates from the entorhinal cortex to connected regions. Several studies have found links between amyloid and tau, suggesting that amyloid pathology is a precursor for tau pathology and influences the distribution of neurofibrillary tangles in the brain (Price and Morris, 1999; Musiek and Holtzman, 2012; Jack et al., 2013). Tau itself has been found to be strongly correlated with tissue atrophy and neurodegeneration, making it a predictor for cognitive impairment at later disease stages (Harrison et al., 2019; La Joie et al., 2020).

The consistency of tau's spatiotemporal progression and its confirmed direct correlation with neurodegeneration make it an optimal target for computational modeling. Personalized models of tau pathology could serve as a tool to predict individual disease progression timelines and as simulated controls in clinical trials. In the latter context, the model may be leveraged to predict how tau would develop in a test subject over time without intervention which can then be compared to the actual developments in the test subject with interventions targeting tau aggregation (Congdon and Sigurdsson, 2018). Multiple groups have proposed network diffusion and epidemic spreading models to simulate the spatiotemporal propagation in the brain for pathological proteins in general (Iturria-Medina et al., 2014; Weickenmeier et al., 2019; Garbarino et al., 2021), and for tau in particular (Raj et al., 2012; Torok et al., 2018; Fornari et al., 2019; Vogel et al., 2020) with good qualitative results. Until recently, the only way to measure the distribution of tau in the brain was through postmortem histology or by making assumptions about the relationship between tau and tissue atrophy observed in structural MRI scans (Raj et al., 2012; Torok et al., 2018). The resulting lack of data has posed significant challenges for calibration of computational tau models. However, an emerging molecular imaging technique, positron emission tomography (PET), now enables us to track the distribution of hyperphosphorylated tau in the brain *in vivo* (Johnson et al., 2016; Villemagne et al., 2018). As the technique is maturing, the amount of available data is growing steadily, allowing us to computationally comprehend the tau pathology in individual subjects over time and use this data for model calibration.

In a recent study, we have shown that we can successfully fit a network diffusion model based on a weighted Laplacian graph of the axonal connectome to longitudinal tau PET data of 46 subjects using a deterministic optimization approach (Schäfer et al., 2020). With tau PET becoming a more established component of longitudinal imaging studies, the amount of available data is steadily increasing, setting the ground for data-driven modeling techniques. Here we use Bayesian hierarchical modeling (Peirlinck et al., 2019) to calibrate the same network diffusion model to longitudinal imaging data from 76 subjects from the Alzheimer's Disease Neuroimaging Initiative (ADNI, 2020). Introducing this probabilistic approach to replace our previous deterministic optimization allows us to account for potential uncertainties in image acquisition and processing, and at the same time, quantify the uncertainty in our model calibration. Identifying the uncertainty in our model parameters is essential to determine the accuracy of our personalized model predictions. If clinical scientists and study designers are to use our model, it is crucial to quantify the accuracy of the simulation. Only then can they determine for which subjects the disease course can be confidently inferred from the available data and for which subjects additional data may be needed to make accurate enough projections. It may also inform them at which time points to acquire additional data to most efficiently improve model accuracy. The hierarchical structure we chose here to represent our model parameters on group and subject levels, will help us gain a better understanding of variability and commonalities of tau pathology between subjects.

## 2. Materials and Methods

**Figure 1:**
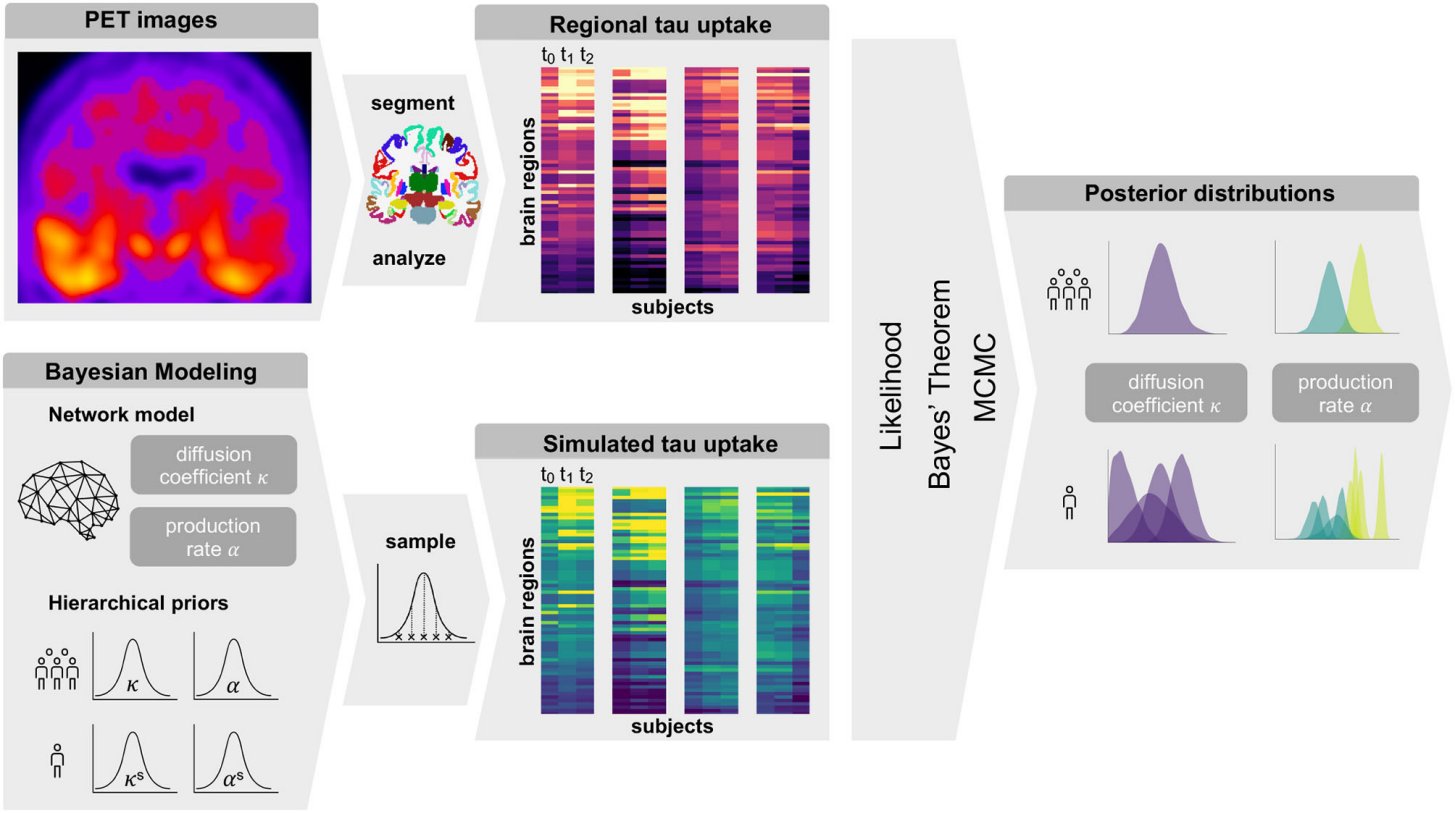
Summary of Methods. Summary of our workflow including PET image analysis and Bayesian modeling to obtain personalized posterior distributions of two model parameters describing the tau pathology in the examined group of subjects.

Figure 1 gives an overview of our methods. In summary, we obtain regional tau uptake values from longitudinal tau PET images through a process of image registration, segmentation, and region of interest analysis. We assume that the propagation of misfolded tau in the brain can be described by a network diffusion model characterized by two model parameters, diffusion coefficient and production rate. After defining weakly informative prior distributions for those model parameters we use a Markov Chain Monte Carlo algorithm to smartly sample from the priors. Inserting the sampled parameters into our model and comparing the resulting simulated tau uptake with the observed data then allows us to rate each sample based on its likelihood and apply Bayes' theorem to determine the posterior distributions of most likely parameter values for each subject.

### 2.1. Network Diffusion Model

**Figure 2:**
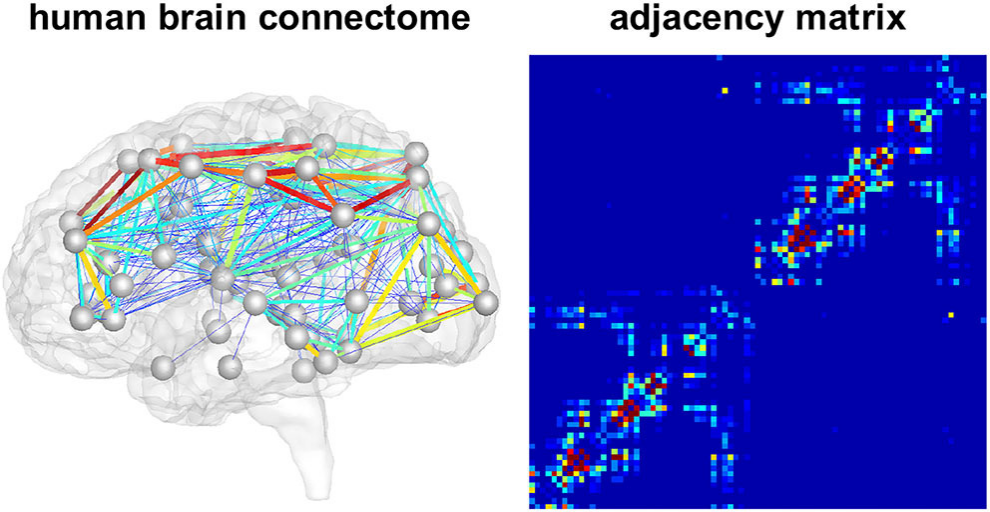
Brain network model. Connectivity-weighted brain network and corresponding adjacency matrix. Colors represent the connection strength between two regions. Connectivity is moderate to strong within the two brain hemispheres while there are only few and weak connections between hemispheres.

We model the accumulation and propagation of hyperphosphorylated tau in the brain's connectome as a diffusion problem on a weighted, undirected graph G with *N* nodes, representing different brain regions, and *E* edges, representing axonal connections between those brain regions. We use the Budapest Reference Connectome v. 3.0 (Szalkai et al., 2017) to obtain the graph G from processed diffusion tensor imaging data of 418 healthy subjects collected through the Human Connectome Project (McNab et al., 2013). From the original graph with *N* = 1015 nodes, we create a reduced graph with *N* = 83 nodes representing 83 cortical and subcortical brain regions. The edge weights of the network are defined by the number of fibers *n*_*ij*_ detected along the respective edge between the pair of nodes *i* and *j*, divided by the fiber length *l*_*ij*_ along this edge averaged across all 418 brains. The adjacency matrix *A*_*ij*_ of the graph, containing the edge weights for all connections, is thus computed as *A*_*ij*_ = *n*_*ij*_/*l*_*ij*_. The resulting network and its adjacency matrix are illustrated in Figure 2, showing a small number of strong and medium connections within and between the lobes of each hemisphere and only few connections between hemispheres.

We characterize the aggregation and spread of pathological tau within the brain connectome as a nonlinear reaction-diffusion problem governed by the Fisher-Kolmogorov equation (Fisher, 1937; Kolmogorov et al., 1937). This equation describes how the concentration of misfolded protein *c* evolves over time based on the assumption that tau pathology develops in a prion-like fashion (Jucker and Walker, 2011; Fornari et al., 2019, 2020).

(1) $$\begin{array}{c} \frac{\text{d}c}{\text{d}t}=\nabla \cdot (\text{D}\cdot \nabla c(t))+\alpha c(t)[1-c(t)], \end{array}$$

Here, **D** denotes the diffusion tensor, which determines the speed and directionality of corruptive tau seed propagation, and *α* the local production rate, which captures the processes of protein production, clearance and conversion from healthy to unhealthy seeds (Fornari et al., 2019). In order to apply the diffusion model to our brain network, we discretize Equation (1) on the weighted graph G. This leads to a discretized diffusion equation expressing for each node of the network *i* = 1, …, *N* the change in nodal concentration of misfolded protein *c*_*i*_ as

(2) $$\begin{array}{c} \frac{\text{d}c_{i}}{\text{d}t}=-\kappa \sum_{j=1}^{N}L_{ij}c_{j}(t)+\alpha \;c_{i}(t)[1-c_{i}(t)]. \end{array}$$

Equation (2) contains two model parameters, *κ* and *α*, which we can calibrate to individual patient data to reflect differences in disease dynamics across individuals. The diffusion coefficient *κ* determines the transport rate of misfolded protein between two regions and *α* the production or clearance of pathological protein at each node. We assume these model parameters to be identical at all nodes *i* = 1, …, *N*, but different between individuals. The weighted graph Laplacian *L*_*ij*_ summarizes the connectivity of the graph. Its diagonal terms contain information about how much protein diffuses out of node *i* into other nodes *j* and its non-diagonal terms describe how much protein enters node *i* from all other nodes *j*. The Laplacian is a square matrix constructed by subtracting the adjacency matrix *A*_*ij*_ from the degree matrix *D*_*ii*_,

(3) $$\begin{array}{c} L_{ij}=D_{ij}-A_{ij}. \end{array}$$

The degree matrix *D*_*ii*_ is a diagonal matrix with each entry representing the sum of elements along a row of the adjacency matrix *A*_*ij*_,

(4) $$\begin{array}{c} D_{ii}=\text{diag}\sum_{j=1,j\neq i}^{N}A_{ij}. \end{array}$$

### 2.2. Image Data

We use longitudinal imaging data from the Alzheimer's Disease Neuroimaging Initiative (ADNI) ADNI (2020) to initialize and calibrate our model. From the database, we select 76 subjects with at least three consecutive tau PET scans, which were acquired on average 1 year (1.07±0.31) apart. This group contains a variety of clinical diagnoses, 31 subjects are diagnosed as cognitively normal, 15 with significant memory concern, 28 with mild cognitive impairment, and two with clinically confirmed Alzheimer's disease. Previously evaluated *β*-amyloid PET images identify 46 subjects as amyloid positive (Landau et al., 2013), meaning the average measured amyloid concentration in their brain exceeds a certain threshold value. We conduct our analysis blind to clinical diagnosis, but take amyloid status into account in our model structure.

All acquired AV1451-PET scans were processed according to standard ADNI protocols (ADNI, 2020). For each subject, we co-register the PET images to a corresponding high resolution T1 weighted magnetic resonance image (MRI) which we segmented into 68 cortical and 45 subcortical regions according to the Desikan-Killiany atlas (Desikan et al., 2006) using FreeSurfer (FreeSurfer, 2020). We use this segmentation to compute regional tracer uptake values from the PET images for the same 83 regions represented in our network model. We normalize these regional uptake values with respect to the uptake in the inferior cerebellar gray matter, which serves as our reference region, in order to gain regional standardized uptake value ratios (SUVR). Since PET recordings in subcortical regions and the hippocampus are known to be contaminated by off-target binding in the choroid plexus and nearby vascular structures (Lowe et al., 2016; Marquié et al., 2017; Lemoine et al., 2018), we focus our model calibration on the remaining 66 cortical regions.

Our network diffusion model delivers regional normalized tau concentrations *c*^sim^, between zero, indicating that no misfolded protein is present, and one, indicating that a maximum amount of misfolded protein is present, 0 ≤ *c*^sim^ ≤ 1. To compare simulated with observed protein concentrations, we need to map the tau PET standardized uptake value ratios into the same zero-to-one interval. To this end, we identify a lower threshold for tau positivity by fitting a Gaussian mixture model with two components to the cumulative raw tau PET data *c*^raw^ from all subjects, time points, and regions. Assuming that many of the included regions must be free from pathological tau, this allows us to determine the minimum raw PET value that should be considered positive. We declare all values below to this threshold of *c*^raw^ = 1.1 to be zero and normalize the remaining raw values such that 0 ≤ *c*^pet^ ≤ 1.

### 2.3. Hierarchical Bayesian Inference

**Figure 3:**
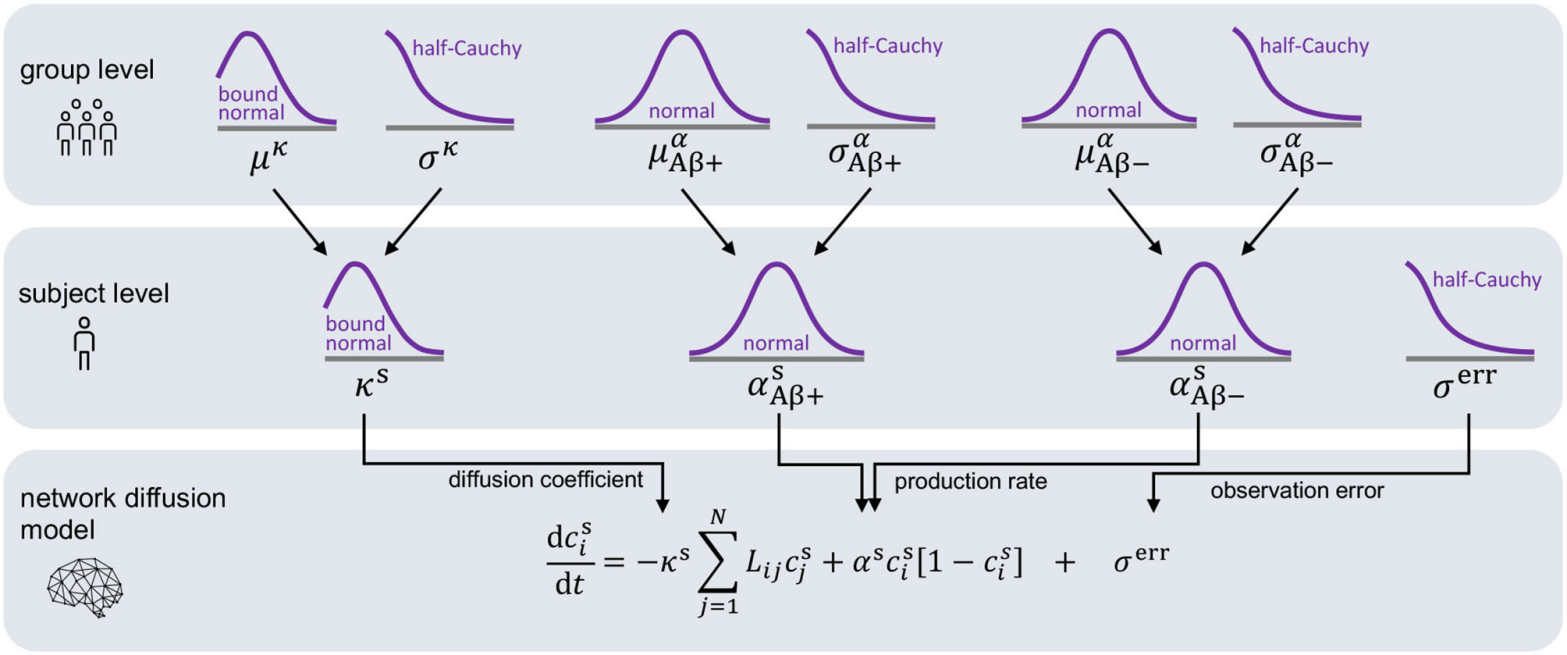
Hierarchical Bayesian inference. Hierarchical structure and prior assumptions for Bayesian inference approach.

For each subject, we infer a personalized diffusion coefficient *κ*^s^ and protein production rate *α*^s^ most accurately reproducing the image data and quantify the uncertainty in our calibration using Bayesian inference. For each subject, we set the initial conditions of our model to the tau uptake values measured in the baseline PET scan $c^{\text{sim}}\left(t=0\right)=c^{\text{pet}}\left(t_{\text{0}}\right)$. Starting from this initial distribution of tau, Bayesian inference allows us to find the parameters that, when inserted into the model, minimize the difference between the model predictions $c^{\text{sim}}\left(t_{\text{i}}\right)$ and the longitudinal tau PET data $c^{\text{pet}}\left(t_{\text{i}}\right)$ for each subject. The timepoints *t*_i_ (*i* = 1, …, *M*) for model evaluation are dictated by the timepoints of PET scan acquisition, with the number of follow-up scans *M* ranging from two to four depending on data availability for each respective subject. To define the prior distributions for our Bayesian inference, we employ the hierarchical structure illustrated in Figure 3. The hierarchical approach allows us to gain personalized posterior distributions while taking into account commonalities between subjects (Gelman and Hill, 2006). Specifically, we assume that the personalized diffusion coefficient *κ*^s^ is represented by a normal distribution bounded to positive values. Additionally, we propose that the hyperparameters *μ*^*κ*^ and *σ*^*κ*^, representing mean and standard deviation of this bounded normal distribution, are drawn from common hyperdistributions for all subjects. To account for potential deviations in pathology based on the subjects' amyloid status, we assume that the personalized production rate, $\alpha_{\text{A}\beta +}^{\text{s}}$ or $\alpha_{\text{A}\beta -}^{\text{s}}$, is drawn from a different normal distribution depending on whether the subject has been identified to be amyloid positive or negative. To account for similarities across subjects within one amyloid status group, we postulate that the hyperparameters $\mu_{\text{A}\beta +}^{\alpha }$ and $\sigma_{\text{A}\beta +}^{\alpha }$ are drawn from common hyperdistributions for all amyloid positive subjects, while the hyperparameters $\mu_{\text{A}\beta -}^{\alpha }$ and $\sigma_{\text{A}\beta -}^{\alpha }$ are drawn from common hyperdistributions for all amyloid negative subjects.

We postulate that the likelihood between the time-dependent PET imaging data $\hat{D}\left(t\right)$ and our model predictions *D*(*t*, ***ϑ***, ***φ***) is normally distributed around the modeled values with a width of σ^err^.

(5) $$\begin{array}{c} p(\hat{D}(t)|\vartheta ,\varphi )\sim \text{Normal}(\text{mean}=D(t,\vartheta ,\varphi ),\text{width}=\sigma^{\text{err}}). \end{array}$$

To complete our statistical model in a Bayesian setting, we select weakly informative priors for our set of model parameters $\vartheta =\left\{\kappa^{\text{s}},\alpha_{\text{A}\beta +}^{\text{s}},\alpha_{\text{A}\beta -}^{\text{s}}\right\}$ and our set of hyperparameters $\varphi =\left\{\mu^{\kappa },\sigma^{\kappa },\mu_{\text{A}\beta +}^{\alpha },\sigma_{\text{A}\beta +}^{\alpha },\mu_{\text{A}\beta -}^{\alpha },\sigma_{\text{A}\beta -}^{\alpha }\right\}$ as summarized in Table 1.

**Table 1:** Hierarchical Bayesian inference.

**Parameter**	**Distribution**
*μ*^*κ*^	BoundNormal(> 0,1,20)
*σ*^*κ*^	HalfCauchy(*β* = 1)
κ^s^	BoundNormal(> 0,*μ*^*κ*^,*σ*^*κ*^)
$\mu_{\text{A}\beta +}^{\alpha }$	Normal(0,2)
$\sigma_{\text{A}\beta +}^{\alpha }$	HalfCauchy(*β* = 1)
$\alpha_{\text{A}\beta +}^{\text{s}}$	Normal($\mu_{\text{A}\beta +}^{\alpha }$,$\sigma_{\text{A}\beta +}^{\alpha }$)
$\mu_{\text{A}\beta -}^{\alpha }$	Normal(0,2)
$\sigma_{\text{A}\beta -}^{\alpha }$	HalfCauchy(*β* = 1)
$\alpha_{\text{A}\beta -}^{\text{s}}$	Normal($\mu_{\text{A}\beta -}^{\alpha }$,$\sigma_{\text{A}\beta -}^{\alpha }$)
*σ*^err^	HalfCauchy(*β* = 1)

*Prior distributions for the personalized diffusion coefficient and its hyperparameters, the personalized production rate and its hyperparameters, and the width of the likelihood*.

Finally, we compute the posterior distributions $p\left(\vartheta ,\varphi |\hat{D}\left(t\right)\right)$ for the model parameters ***ϑ*** and hyperparameters ***φ*** using Bayes' theorem,

(6) $$\begin{array}{c} p(\vartheta ,\varphi |\hat{D}(t))=\frac{p(\hat{D}(t)|\vartheta ,\varphi )p(\vartheta ,\varphi )}{p(\hat{D}(t))}, \end{array}$$

with *p*(***ϑ***) denoting the prior distributions from Table 1. Since we cannot solve for the posterior distributions analytically, we adopt approximate-inference techniques to calibrate our model to the imaging data. Specifically, we use the No-U-Turn sampler (NUTS) (Hoffman and Gelman, 2014), a type of Hamiltonian Monte Carlo algorithm implemented in the python package PyMC3 (Salvatier et al., 2016) to numerically approximate the posterior distributions. We run two chains with 1,600 tuning samples and 2,000 post-tuning samples each. After convergence of the posterior distributions, we draw 4,000 posterior predictive samples of different parameter combinations which allow us to quantify the uncertainty on the inferred parameters. Additionally, we sample from the posterior distributions to predict the evolution of tau in three brain regions of interest in 35 subjects with a positive production rate. Specifically, we predict how the tau concentration is projected to change over the next 30 years in the entorhinal cortex (EC), the middle temporal gyrus (MTG) and the superior temporal gyrus (STG). Post mortem histological studies have shown that these regions are affected by hyperphosphorylated tau and neurofibrillary tangles at different disease stages, the entorhinal cortex falling into Braak stage II, the middle temporal gyrus into Braak stage IV and the superior temporal gyrus into Braak stage V (Braak et al., 2006). By propagating the uncertainty from the parameter inference through the posterior predictions, we gain an ensemble of forecasts enabling us to determine the credible intervals around our predictions.

## 3. Results

### 3.1. Posterior Distributions

**Figure 4:**
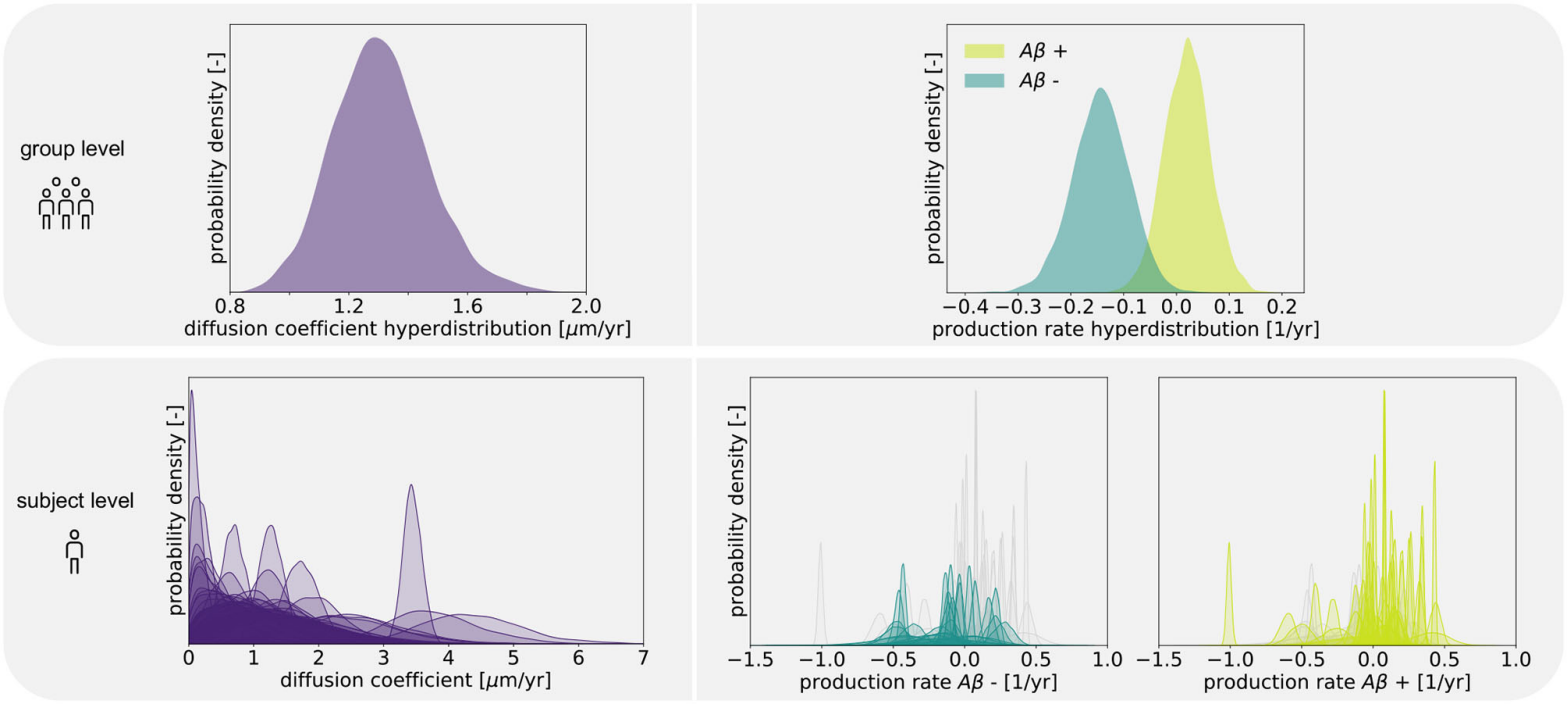
Posterior distributions. Posterior distributions for diffusion coefficient and protein production rate on group and subject levels. Subject-wise distributions for the production rate are depicted in light and dark green in separate plots based on amyloid status. In each of those plots, the individual distributions associated with the other amyloid status group are depicted in light gray for comparison.

Figure 4 shows the posterior distribution density plots for the personalized model parameters *κ*^s^, $\alpha_{\text{A}\beta +}^{\text{s}}$ and $\alpha_{\text{A}\beta -}^{\text{s}}$, as well as for the hyperparameters *μ*^κ^, $\mu_{\text{A}\beta +}^{\alpha }$ and $\mu_{\text{A}\beta -}^{\alpha }$. The personalized diffusion coefficient, characterizing how fast tau spreads along a single connection between two regions, is physically constrained to be positive. We found that this parameter takes on values of up to 4.38 μm/yr. Across all subjects we identified an average diffusion coefficient of 1.304 ± 0.69 μm/yr. The protein production rate can take on positive or negative values, depending on whether clearance or production of pathological protein dominate in a particular subject. Both amyloid groups contain subjects with positive and subjects with negative production rates. However, the density plots of the hyperparameters show that there is a noticeable difference in the group-level mean production rate depending on amyloid status. Subjects with negative amyloid status tend to exhibit a lower protein production rate than subjects with positive amyloid status. We identified an average production rate of −0.143 ± 0.21/yr across all amyloid negative subjects and 0.019 ± 0.27/yr across all amyloid positive subjects. Table 2 summarizes the mean, maximum, and minimum inferred values for all personalized model parameters.

**Table 2:** Posterior distributions.

**Parameter**	**Diffusion coefficient** ***κ***^**s**^	**Production rate** $\alpha_{\text{A}\beta -}^{\text{s}}$	**Production rate** $\alpha_{\text{A}\beta +}^{\text{s}}$
	**[μm/yr]**	**[1/yr]**	**[1/yr]**
**Mean**	1.304	−0.143	0.019
**Std**	±0.69	±0.21	±0.27
**Min**	0.15	−0.49	−1.01
**Max**	4.38	0.27	0.44

*Mean values, standard deviations, maximum and minimum values for personalized model parameters*.

**Figure 5:**
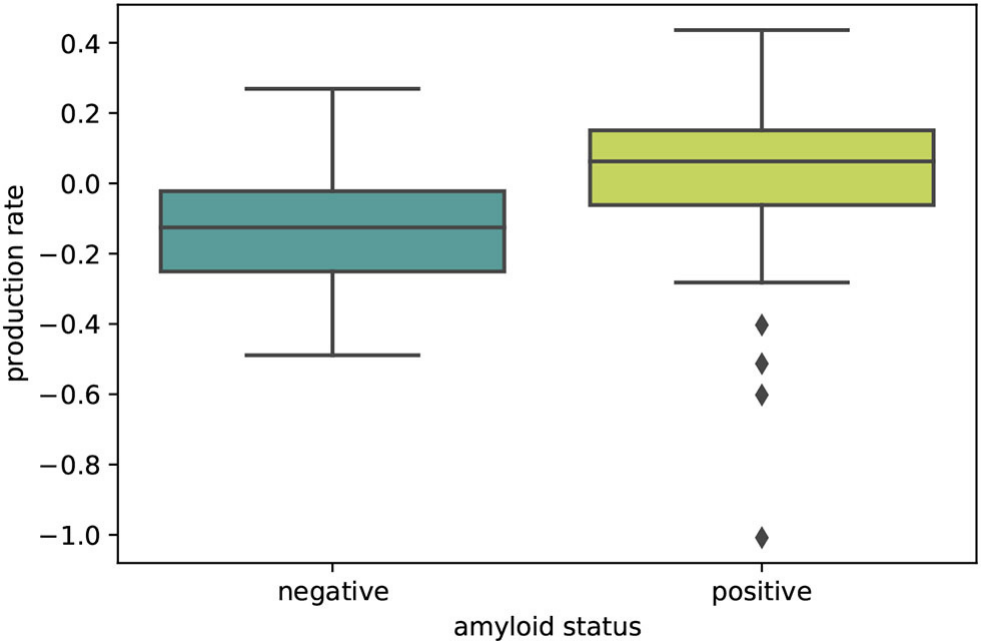
Amyloid status. Boxplot illustrating the distributions of personalized production rates in amyloid negative and amyloid positive subject groups. The difference between the two groups is significant with *p* = 0.0075.

The boxplot in Figure 5 further illustrates the effect of amyloid status on the inferred personalized production rate. When comparing the average production rates associated with amyloid positive and negative groups in an independent *t*-test, we found that the difference is significant with p = 0.0075. While there are some outliers toward negative values in the amyloid positive group, overall the production rate associated with amyloid positive subjects is significantly higher than the production rate associated with amyloid negative subjects. Our results did not show any significant and consistent trends in diffusion coefficients or production rates associated with different clinical diagnoses, e.g., cognitively normal, mild cognitive impairment, or Alzheimer's disease (see Supplementary Figure 1).

### 3.2. Posterior Predictive Modeling

**Figure 6:**
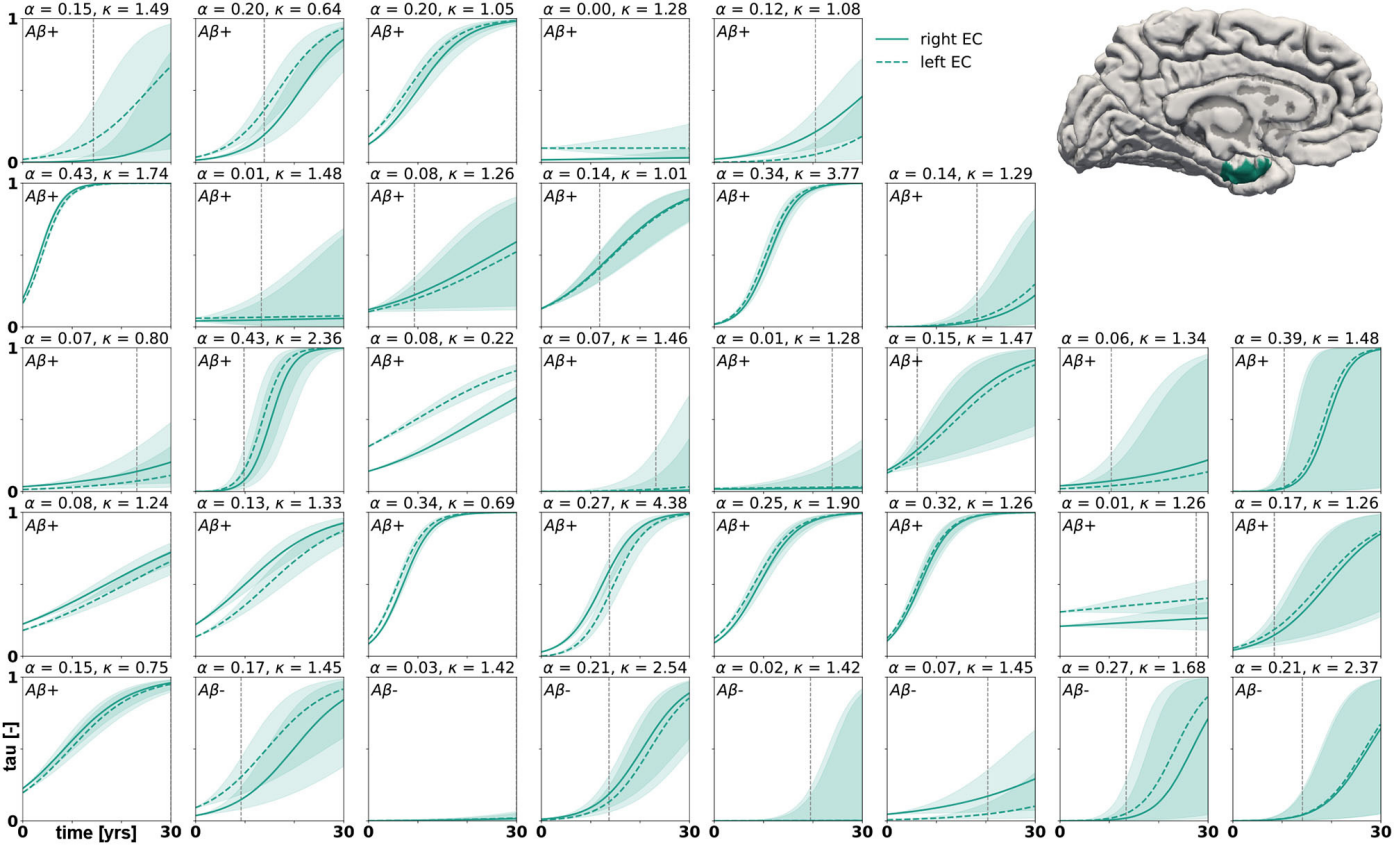
Posterior predictive modeling. Predictions for the change in tau in the entorhinal cortex in 30 years after first tau PET scan for 35 subjects. Shaded areas around the curves represent the 95% credible intervals of the predictions.

**Figure 7:**
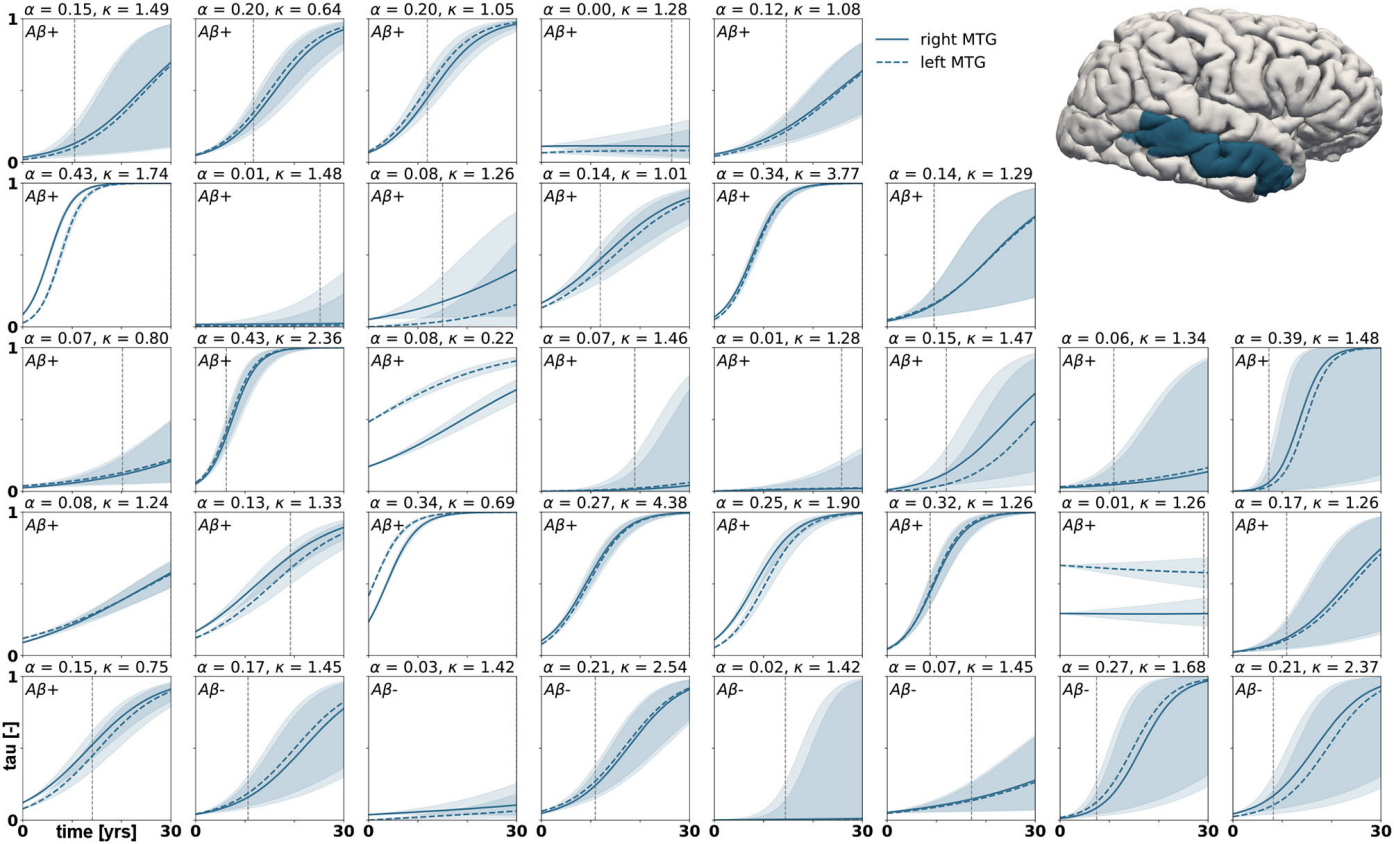
Posterior predictive modeling. Predictions for the change in tau in the middle temporal gyrus in 30 years after first tau PET scan for 35 subjects. Shaded areas around the curves represent the 95% credible intervals of the predictions.

**Figure 8:**
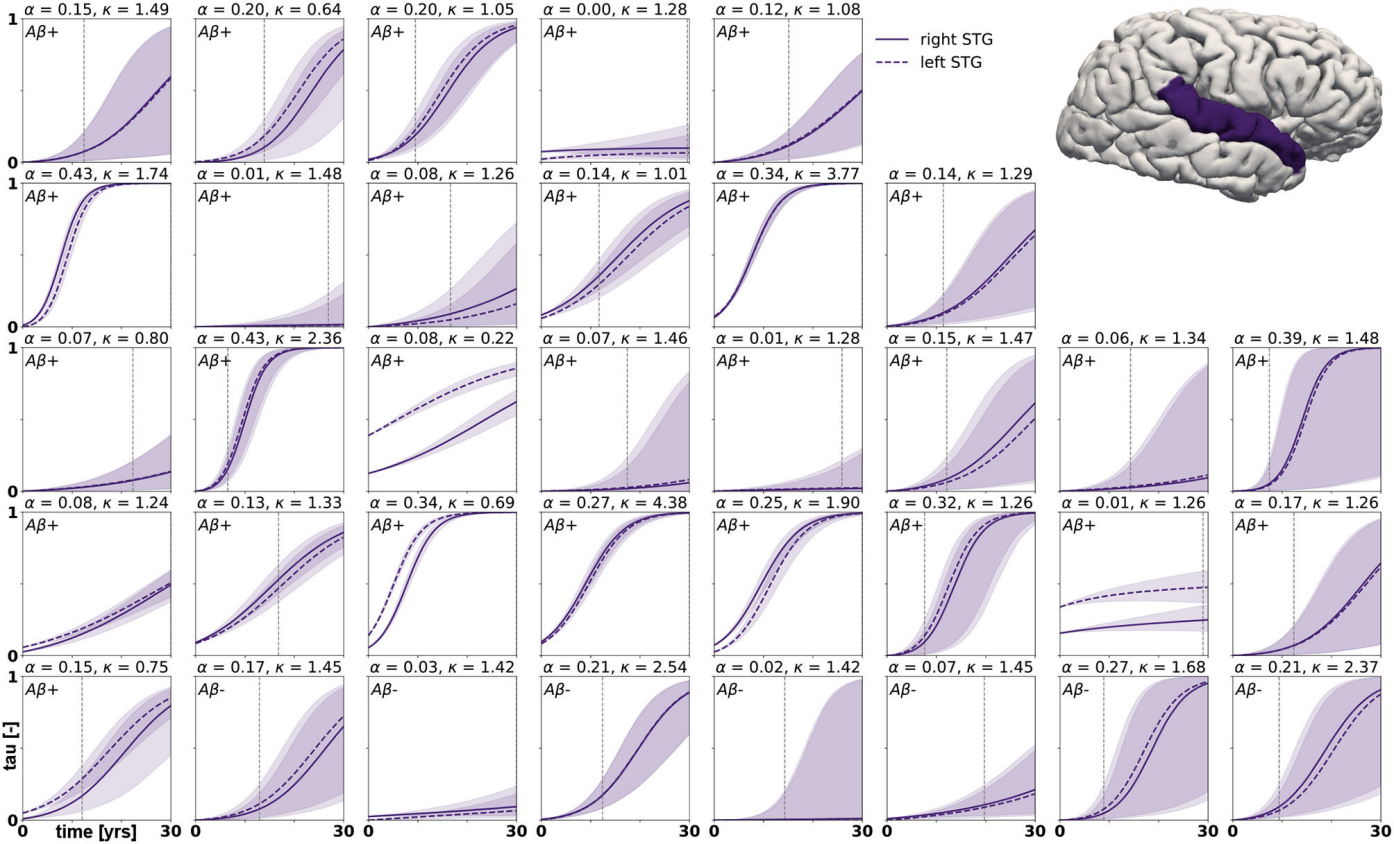
Posterior predictive modeling. Predictions for the change in tau in the superior temporal gyrus in 30 years after first tau PET scan for 35 subjects. Shaded areas around the curves represent the 95% credible intervals of the predictions.

Posterior predictive modeling allows us to propagate the uncertainty from the Bayesian inference process through the model and illustrate its impact on model predictions. Figures 6–8 show our projections for tau evolution over 30 years after the first PET scan in 35 subjects and three different brain regions. The sigmoid like shape of the curves is characteristic for the combined diffusion production equation we use to model the spread of pathological tau and local conversion from healthy to unhealthy proteins. The shaded area around the curves represents the 95% credible interval, quantifying the uncertainty in our predictions as established by the probabilistic approach. Narrow credible intervals indicate high confidence in our predictions. The curves are fairly symmetrical across left and right hemisphere. When comparing the predictions for different subjects within entorhinal cortex (EC), middle temporal gyrus (MTG) and superior temporal gyrus (STG), we can identify a number of subjects for which the credible interval is narrow, confirming high certainty for our predictions. Specifically, there are seven subjects for whom the credible interval does not exceed a width of 0.2 over 30 years in any of the three examined brain regions. For multiple other subjects however, the credible interval is rapidly widening after only a few years. For these subjects, the available imaging data did not yet contain enough information to confidently infer personalized model parameters with our probabilistic approach. In those instances, additional data from PET scans at future time points may improve the prediction certainty. The vertical gray lines in Figures 6–8 indicate the year at which the width of the credible interval exceeds a critical threshold of 0.2. If the goal is to collect additional data to increase confidence in our projections, these time points would be reasonable choices for additional scans. The value of 0.2 was chosen arbitrarily to illustrate how our uncertainty predictions can inform future study design if there is a known confidence requirement for predictions.

## 4. Discussion

In this study we used a probabilistic approach based on hierarchical modeling and Bayesian inference to identify personalized model parameters of a physics-based network diffusion model for misfolded tau propagation. We calibrated our model to longitudinal tau PET data of 76 subjects and created personalized predictions for disease progression over a course of 30 years. Propagating the uncertainty from our parameter search through the posterior predictions allowed us to determine the credibility associated with our predictions for different subjects and brain regions.

We based the structure of our hierarchical model on the assumption that the protein production rate, summarizing the process of healthy protein production, protein clearance and conversion from healthy to misfolded protein, may vary between amyloid positive and amyloid negative subjects. This assumption makes sense in light of the amyloid hypothesis, which identifies amyloid pathology as the primary hallmark of Alzheimer's disease. In fact, tau pathology has been found in medial temporal limbic areas before the appearance of any amyloid plaques (Braak and Del Tredici, 2011). However, these early tau accumulations are usually so small that they can only be detected by immunostaining methods and are rather related to normal aging than to Alzheimer's disease. It has been suggested that, independent from previously existing minor tauopathy, amyloid pathology intensifies and accelerates any existing tauopathy through currently unknown mechanisms (Price and Morris, 1999; Jack et al., 2013). This may allow hyperphosphorylated tau to spread widely across the neocortex (Musiek and Holtzman, 2012; Kevrekidis et al., 2020; Thompson et al., 2020). Our results support the hypothesis that amyloid pathology is a driver for tau pathology. Even though the structure of our probabilistic model does not enforce a difference between the production rate hyperdistributions for amyloid positive and negative groups, we found that misfolded tau production rates were significantly higher in amyloid positive subjects than in amyloid negative subjects.

Across all examined subjects, we identified an average tau diffusion rate of 1.304 μm/yr. *In vivo* experiments in mice determined that healthy tau proteins move as part of the slow component of axonal transport at 0.2–0.4 mm/day in retinal ganglion cell axons (Mercken et al., 1995). There seems to be a strong disconnect between the slow time scale of tau pathology evolution in Alzheimer's disease, which is known to typically stretch over more than a decade (Bateman et al., 2012), and the fast time scale of axonal transport. If misfolded tau spread in the brain at the speed measured for healthy tau, it would easily contaminate the whole brain in just a few months. This scenario appears inconsistent with the slow propagation and discrete stageing of neurofibrillary tangles and neuropathology that has been observed in histopathological (Braak and Braak, 1991) and imaging studies (Jack et al., 2018).

A possible explanation for this discrepancy in time scales could be related to how protein diffusion and production contribute to tau pathology to varying extents depending on the stage of disease. The model we use here to describe the propagation of tau pathology in the brain is based on the hypothesis that misfolded tau contaminates the brain in a prion-like fashion (Jucker and Walker, 2011; Fornari et al., 2020). We assume that hyperphosphorylated tau proteins act as proteopathic seeds that can travel along the axon, leave the cell and be taken up into previously unaffected neurons (Clavaguera et al., 2009; Liu et al., 2012). Additionally, we adopt the hypothesis that misfolded tau seeds replicate and aggregate locally (Iba et al., 2013). Once the chain reaction consisting of the spread of proteopathic seeds and the local multiplication of seeds is initiated, it results in an overall increase of misfolded tau across the brain. However, it is difficult, if not impossible, to determine experimentally which of the two components, diffusion or local production, dominates during different stages of disease.

A recent study aimed at quantifying the chemical kinetics of tau replication and spreading from several modalities of data, including seed amplification assays, histopathology, and tau PET, found that protein replication, not spreading, is the dominant and limiting component of tau accumulation after a certain stage of disease (Meisl et al., 2020). The authors argue that misfolded tau seeds spread very fast early in the disease process, consistent with the fast axonal transport rates known for other proteins. After this initial fast spread, small but significant amounts of proteopathic seeds are already present in numerous brain regions, and further kinetics are largely determined by the local replication and aggregation of those seeds. These findings are consistent with our results, which indicate very low diffusion coefficients. Unless our data were capturing the very beginning of tau pathology, we will not be able to infer the fast transport rates that might determine disease progression initially. Since we use the regional tau distribution from each subject's baseline PET scan instead of an artificial seeding approach, it is common that small amounts of tau are already measured in a majority of the brain regions. The protein production rates we identified for our subject sample are comparable to the average replication rate of 0.14/yr reported in the study above (Meisl et al., 2020).

We computed personalized 30-year predictions of tau evolution in three different brain areas, the entorhinal cortex, the middle temporal gyrus and the superior temporal gyrus. Since there is a well established correlation between tau distribution and neurodegeneration, these predictions not only contain information about the amount of protein in these areas, but also provide important clinical insight into when certain brain functions might be affected. A recently conducted study compared tau PET distribution at baseline visit to the amount and distribution of atrophy detected between baseline and follow-up visit (La Joie et al., 2020). It was found that tau is a strong predictor for regional atrophy presenting around 15 months after the PET scan. In the healthy adult brain, the neurons in the entorhinal cortex provide a number of functionalities, but are mainly thought to be responsible for spatial memory and spatial association tasks (Kerr et al., 2007; Van Strien et al., 2009; Kuruvilla and Ainge, 2017). If this area atrophies after serious invasion of misfolded tau protein, these functions may be impaired or lost. The middle temporal gyrus, which is part of the inferior temporal lobe, has been suggested to play a central role in visual learning and memory (Buckley et al., 1997) and lesions in this region may cause object and face recognition deficits (Purves et al., 2001). The superior temporal gyrus contains the auditory cortex and is involved in speech and auditory processing (Gernsbacher and Kaschak, 2003) as well as social cognition (Adolphs, 2003; Bigler et al., 2007). The quantified uncertainty on disease progression showcased in Figures 6–8 indicates the credibility associated with each subject's prediction specifically, taking into account the behavior of the whole cohort. This framework may provide an interesting tool for clinical prognosis, informing clinical practitioners and caregivers when cognitive symptoms related to loss of the functions above maybe be expected in a certain patient. It may also provide a new means to assess the optimal time for a follow-up scan, smartly maximizing prognosis credibility while minimizing the number of scans performed.

This study comes with a number of limitations. First, the amount of longitudinal tau PET data available today is limited. The small sample size and limited follow-up data included in our study result in large credible intervals and reduced confidence in the model parameters. As tau PET becomes a more established technique in longitudinal imaging studies over the next years, more data will naturally become available, allowing us to constantly improve our hierarchical model, as Bayesian methods are inherently tailored to analyzing data that are continuously updated in time. Adding more subjects to our data set will further increase the learning effect we achieve through the hierarchical structure, which will in turn increase the credibility of all personalized predictions. Larger sample sizes of data in the future will also allow us to explore more complex models, e.g., models introducing regionally varying protein production rates based on local gene expression (Grothe et al., 2018), without the risk of overfitting. Second, we use the same anatomical brain network to compute tau spreading in all subjects. This network was extracted from averaged diffusion tensor images of over 400 brains. In reality, the connectivity is different in every brain, potentially affecting the diffusion dynamics observed here. We attempt to surmount this issue by introducing the diffusion coefficient as a personalized parameter. It would be reasonable to assume that the transport rate of misfolded tau along the axon is a biological parameter that is similar in all brains. However, by allowing this parameter to vary between subjects, we provide the option to scale the adjacency matrix and thereby introduce variations in connection strength for the otherwise non-personalized network. In the future, we plan to surpass the potential over-generalization that using an average network introduces by extracting personalized connectomes for each subject from diffusion tensor images when available. Another consideration for future studies is to correct the weighting of connections in our network for the varying surface areas between brain regions. Additionally, it could be of interest to compare the performance of our model on the structural connectome with its performance on other reference networks, and thus test the hypothesis that misfolded tau spreads along the axonal network. Third, since the ADNI data base only provides one tau PET scan for each time point, this study does not explicitly take into account uncertainties arising from imaging protocols. However, since we expect this error to be Gaussian, it is partially accounted for by the stochastic nature of the observation error in our Bayesian inference framework. In contrast to deterministic optimization algorithms, our probabilistic approach inherently accounts for observation errors through the likelihood width.

The approach we used here is optimal for understanding the applicability of the physics-based network diffusion model to longitudinal brain imaging data and for quantifying the range of model parameters presented in this data. Additionally, the Bayesian inference framework inherently provides information on the uncertainty in our model, intrinsically informing us on model applicability. In the future, we will expand our model to a more predictive approach using a combination of deep learning, Bayesian inference, and physics-based modeling, with the goal to create personalized predictions of tau spreading dynamics from a single baseline PET scan. Furthermore, we will explore a coupled model of tau pathology and resulting tissue atrophy (Schäfer et al., 2019) calibrated to longitudinal tau PET and structural MRI.

## 5. Conclusion

We presented a probabilistic approach to calibrate the parameters of a physics-based network diffusion model to longitudinal tau PET data. We obtained posterior probability distributions for two personalized model parameters, the diffusion coefficient and the protein production rate, using Bayesian inference combined with a hierarchical prior structure. This approach allowed us to identify the characteristics of tau propagation for each individual subject while taking into account expected commonalities between subjects. We inferred an average diffusion coefficient of 1.304±0.69 μm/yr, a protein production rate of 0.019±0.27/yr for the amyloid positive group, and a production rate of −0.143±0.21/yr for the amyloid negative group. The significantly higher tau production rate associated with the presence of amyloid-β supports the hypothesis that amyloid pathology drives tau pathology. The small magnitude of our inferred diffusion coefficients is inconsistent with experimentally identified axonal transport rates for healthy tau, but consistent with the slow disease progression known for Alzheimer's disease. Extrapolating our model based on the posterior distributions of model parameters allowed us to create personalized predictions of tau evolution in three brain regions associated with distinct cognitive functions. These predicitons and associated credibility intervals may serve as a tool to estimate the timeline of regional tau pathology and function-specific cognitive impairment in individual patients. Our findings could serve as simulated controls in therapeutic trials or as a means to smartly schedule follow-up PET scans that most benefit model prediction certainty.

## Data Availability Statement

Publicly available datasets were analyzed in this study. This data can be found here: http://adni.loni.usc.edu/.

## Author Contributions

AS was responsible for conception and design of the study, implementation of the model, analysis and interpretation of results, and draft of the manuscript. MP, KL, and EK contributed to and guided study conception and design and provided critical revision of the manuscript for intellectual content. All authors approved the final version of the article to be published.

## Conflict of Interest

The authors declare that the research was conducted in the absence of any commercial or financial relationships that could be construed as a potential conflict of interest.
