# Supplementary material for: Bayesian Physics-Based Modeling of Tau Propagation in Alzheimer's Disease
Source: Front Physiol. 2021 Jul 16;12:702975. doi: 10.3389/fphys.2021.702975 (PMC8322942; doi:10.3389/fphys.2021.702975)
Supplement: Supplementary file 1 [file Data_Sheet_1.pdf]

Supplementary Material

1 SUPPLEMENTARY FIGURES

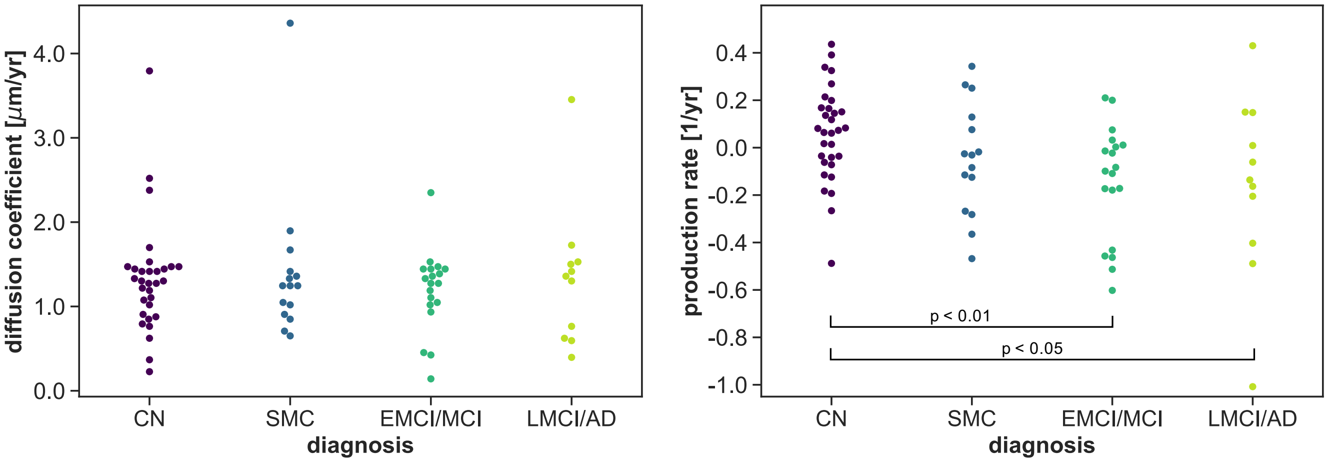

Figure S1. Distribution of personalized model parameters stratified by clinical diagnosis: CN = cognitively normal, SMC = significant memory concern, EMCI = early mild cognitive impairment, MCI = mild cognitive impairment, LMCI = late mild cognitive impairment, AD = Alzheimer's disease. Subjects diagnosed with EMCI and LMCI were grouped together with MCI, and AD subjects to create reasonable sample sizes. An independent t-test showed no significant inter-group differences in diffusion coefficients. Differences in production rates associated with CN vs. EMCI/MCI groups and CN vs. LMCI/AD groups are statistically significant. Due to the small group sizes and large intra-group variations, these results should be interpreted with caution.

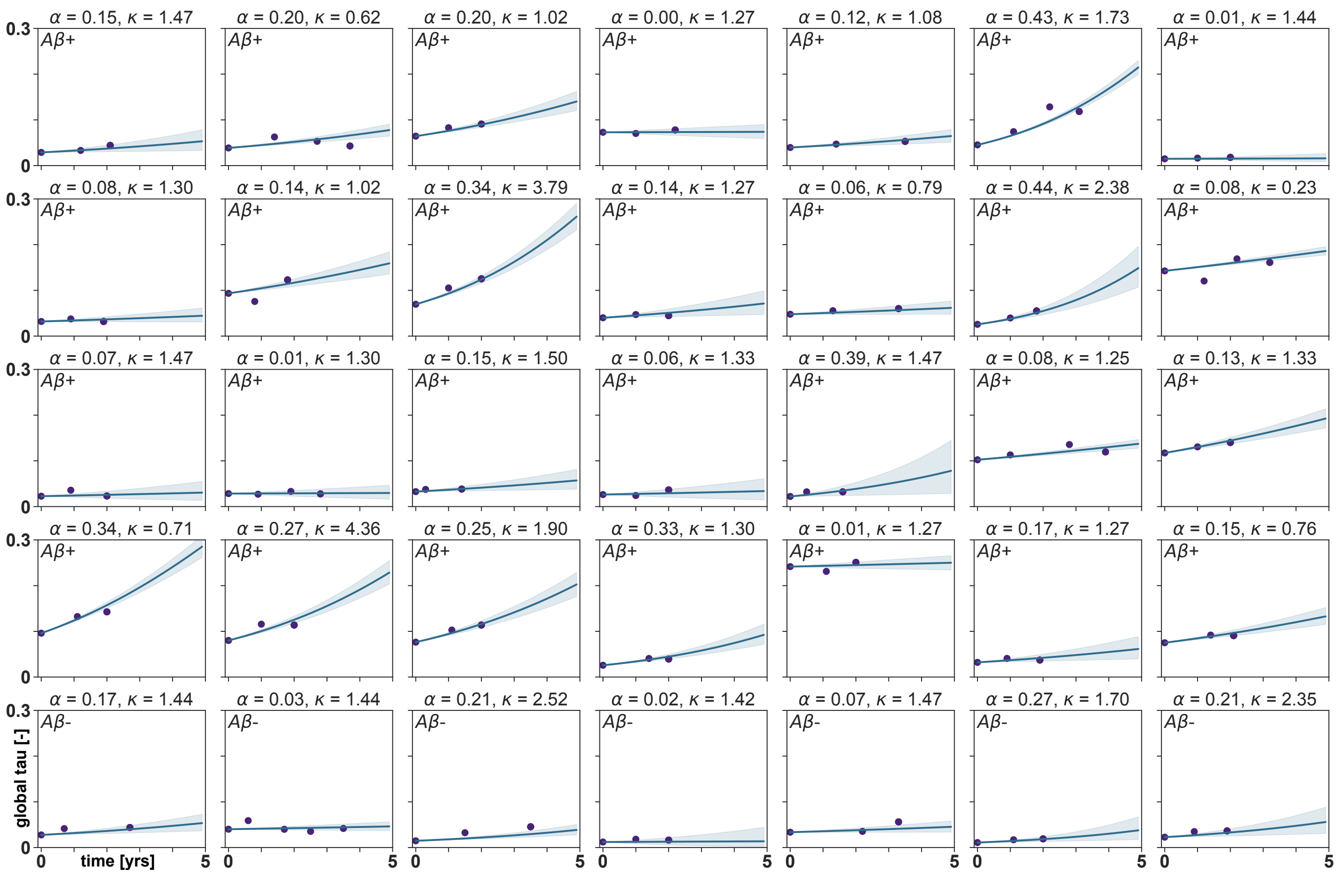

Figure S2. Measured and predicted global average tau load during the first five years after baseline scan. The figure includes all 35 subjects for which we identified a positive protein production rate. Datapoints indicate the tau concentration averaged across all brain regions as measured in the baseline and follow-up PET scans for each subject. Solid lines represent our optimized model solution with shaded areas illustrating the 95 % credible intervals.
